# Family Medicine in Iran: Facing the Health System Challenges

**DOI:** 10.5539/gjhs.v7n3p260

**Published:** 2014-11-30

**Authors:** Reza Esmaeili, Mohammad Hadian, Arash Rashidian, Mohammad Shariati, Hossien Ghaderi

**Affiliations:** 1 Department of Health Economics, School of Health Management and Information Sciences, Iran University of Medical Sciences, Tehran, Iran; 2 Department of Health Management and Economics, School of Public Health, Tehran University of Medical Sciences, Tehran, Iran; 3 Community Medicine Department, School of Medicine, Tehran University of Medical Sciences, Tehran, Iran

**Keywords:** general practice, primary health care, health reform

## Abstract

**Background:**

In response to the current fragmented context of health systems, it is essential to support the revitalization of primary health care in order to provide a stronger sense of direction and integrity. Around the world, family medicine recognized as a core discipline for strengthening primary health care setting.

**Objective:**

This study aimed to understand the perspectives of policy makers and decision makers of Iran’s health system about the implementation of family medicine in Iran urban areas.

**Materials/Patients and Methods:**

This study is a qualitative study with framework analysis. Purposive semi-structured interviews were conducted with Policy and decision makers in the five main organizations of Iran health care system. The codes were extracted using inductive and deductive methods.

**Results:**

According to 27 semi-structured interviews were conducted with Policy and decision makers, three main themes and 8 subthemes extracted, including: The development of referral system, better access to health care and the management of chronic diseases.

**Conclusion:**

Family medicine is a viable means for a series of crucial reforms in the face of the current challenges of health system. Implementation of family medicine can strengthen the PHC model in Iran urban areas. Attempting to create a general consensus among various stakeholders is essential for effective implementation of the project.

## 1. Introduction

Thirty seven years after the Alma Ata Declaration, policy-makers come to believe more and more that it is essential to support the revitalization of primary health care (PHC) in order to restore more sense of direction and integrity in the current fragmented context of health systems. They also believe investing in PHC-based reforms lead to the sustainable development of health system and leads to the achievement of better and more rationally distributed outcomes (WHO, 2008). This valuable point has led health system policy-makers across the world to seek an effective model for providing PHC (Montegut et al., 2004). The principles of Family Medicine (FM) have turned this discipline into a constructive and integrate part of PHC around the world (Montegut et al., 2004; World Bank Group, 2007). Various studies demonstrated the association of FM with better health outcome even in states with poor equality in health (Maeseneer & Flinkenflögel, 2010; Voort et al., 2012). For this reason FM has become a core discipline of providing PHC with a long history in developed health systems and being one of the major reforms in developing health systems in the recent decades (Saltman et al., 2005, Kringos et al., 2010; Masic et al., 2014; Oleszczyk et al., 2012).

FM has been a major policy-making issue in Middle Eastern countries. But its true potential is yet to be realized as there are many obstacles in the path to its implementation (Abyad et al., 2007). Across this region, FM was adopted in Turkey in 1996 as the most comprehensive model for PHC, and was implemented from 2003 to 2008 across 13 provinces and had developed throughout the country by the end of 2010 (Kringos et al., 2011). In Iran, alongside the development of PHC, FM also became a strategic intervention for the restructuring and developing of the health system during the last decade (Gressani et al., 2007; Moghaddam et al., 2013).

### 1.1 Iran Context

In Iran, the basis of PHC is the health networks formulated and developed 1979-1984. This structure received full public funding and focused on the social participation and inter-sectoral collaboration and aimed to provide easy public access to services. The wide expansion of the network all over the country, particularly across rural areas, brought about huge achievements such as a dramatic reduction in infant, mother and newborn mortalities (Shadpour, 2000; Takian et al., 2013). In recent years, this system proved to lack the necessary flexibility for meeting the emerging needs of the community. In response to this weakness, the government introduced health reforms such as the introduction of the FM and rural health insurance (Takian et al., 2011). Accordingly the family medicine and the referral system were initiated in 2005 for rural population in the setting of the health network, and then expanded to cities with populations below 20,000 (Rashidian et al., 2013). The reinforcement of the rural health network with the FM improved public access to health care in villages and small cities while the absence of this integrated model in urban areas created the limitations in accessibility and in the extent of services (Takian et al., 2011; Moghadam et al., 2012). Iran’s health system is now running a pilot project across two the country’s provinces (Fars and Mazandaran) with the ultimate goal of implementing the family physician project for its entire urban population. Although anticipated that it’s countrywide implementation be the cornerstone of vast changes in Iran’s health system (Majdzadeh, 2012), but there are still difficulties in generalization of the project and requires a general consensus among stakeholders. The present study aimed to explore the perspectives of policy-makers and decision-makers in major organizations of Iran health system about the role of family physician in the strengthening of Iran’s health system for facing the current challenges.

## 2. Materials and Methods

This is a qualitative study. Data collection involved semi-structured interviews with policy-makers and decision-makers in five main organizations involved in the design and implementation of the urban family physician project in Iran (Table 1). Necessary arrangements for interviews were made over the phone. One of the researchers (RE) conducted all interviews. He traveled to the pilot provinces in order to capture the views of the local executive directors. Interviews were conducted face-to-face between October 2013 and May 2014 with use of an interview guide. Each interview lasted 50 to 75 minutes and was digitally recorded with participants’ consent. Lastly, interviews were transcribed verbatim in Persian. Collection and initial analysis of the data was concurrent, data collection continued as long as the saturation achieved. We use used framework method for data analysis which specifically developed for analysis of qualitative data in policy-making studies (Rashidian et al., 2008; Gale et al., 2013). We adopted the framework method from Gale et al. (2013) included 7 stages; transcription, familiarization with the interview, coding, developing a working analytical framework, applying the analytical framework, charting data into the framework matrix and interpreting the data. One researcher (RE) performed the transcript. All Members of the research team thoroughly read and re-read each transcript, to become familiar with the interview content. The first Author (RE) coded the first five transcripts. Then, authors met and identified themes that formed the initial analytical framework. We applied the analytical framework to all transcripts; meanwhile we were enthusiasm to capture new codes. The following strategies were applied about four rigor criteria included credibility, dependability, transferability, and confirmability (Tobin and Begley, 2004) respectively. The transcripts with extracted codes sent to a number of participants for feedback about confirmation (Ryan et al., 2007). All researchers with their different backgrounds participated in data analysis and discussion about the editing of the codes (Anney, 2014). We try to report a thick description of our study contextual information (Meyrick, 2006), and we use data triangulation (more than one source of information) in our purposive sampling (Cote & Turgeon, 2005). The overall process review performed using the COREQ 32-item checklist (Tong et al., 2007).

**Table 1:** Participants Chrastristics

		Health Organizations				

		Ministry of Health & Medical Education	Iranian Health Insurance Organization	Medical Universities and Affiliated Research Centers	Iran Medical Council	Executive Directors in Pilot Provinces
Education	MS	-	1	-	-	-
	General Practitioner	2	2	6	1	3
	Specialist	3	-	1	-	2
	PhD	2	-	3	1	-

Gender	Male	7	2	8	2	-
	Female	-	1	2	-	-

Total	7	3	10	2	5

## 3. Results

We analyzed 27 semi-structured interviews were conducted with Policy and decision makers in different health organizations in Iran. With the use of framework method we identified three main themes and 8 sub-themes including: development of referral system, better access to health care, and the management of chronic diseases.

### 3.1 Development of Referral System

There was a strong sense among our participants that the development of the referral path in Iran’s health system is utmost necessity. The participants insisted that the development of referral system is a main mandate of the Iran development plans and family physician is the means whereby this goal can be achieved. They also believed even the family physician project becomes relevant and initiates health system reforms when the referral system is put in place.

“If we are to have a comprehensive program of health system reforms, the referral system is the chief issue that must be on the agenda. And of course, one of the components of the referral system is the family physician…. These are the cases that make this project so urgent in our country”

Many participants depicted that the current place of general practitioners (as the main health human resource) in the country is not one to attract people to general practices or to maintain practitioners’ motivation to keep providing services in this discipline. They recognized that family physician helps general practitioners assume a dominant role in providing health services and welcomed the revival of the role.

“In our country, general practice is in a precarious position. In fact, general practitioners view general practice certificates as stepping stones to something further and the majority seek to complete their GP residency program as quickly as possible to then pursue specialization –a reality that is actually threatening our general practice, which is in fact at the forefront of the exposure of people and their diseases to the health care system. Perhaps the family doctor scheme can revive this status”

The interviewees also stated that specialization has prominent role in people health care seeking behaviors and patients prefer to be visited by specialists even in first contact. Our participants believed this trend imposed expensive and fragmented treatment procedures to patients and they insisted through implementation of family physician, general practice will be as first level contact.

*“People are also specialization-oriented and turn to specialists even for the most minor of diseases and concerns and for consultations for which the general practitioner is fully trained. That is why out of pocket payments have escalated. Secondly, specialized prescriptions have also soared and, unfortunately, the indiscriminate use of MRI, CT scan and specialized tests has become the norm in our country*.

### 3.2 Better Access to Health Care

Participants believed that, in some cases, patients are not properly responded to and are left wandering in search of meeting their needs. They reported that by creating a deeper long-term physician-patient relationship and facilitating the family doctor’s guidance of the patients based on their needs and potentially their referral to health care providers, family medicine can provide the patients with a greater peace of mind.

*“We see that people are confused about the diagnosis of their disease and visit several specialists. This confusion is stressful and imposes huge costs on the household. Family doctors can prevent this confusion*”.

Respondents believed that the implementation of this project will make people’s access to medical consultation easier and that, in the meantime, general health care extends to all urban areas uniformly, especially areas farther away from the center and the gap between the passive nature of PHC providing across urban areas and its widespread active providing across rural areas will be filled.

*“I respect and support organizers of the scheme, because we have patients that truly need to be treated by the system for free”*.

*“As a physician who started work in rural areas in 1994, I could see that all primary health care services were performed promptly and I could see for myself the active care provided for target and sensitive groups, in particular for infants, newborns, pregnant women and the elderly. In the cities, because of both the limited number of health care centers and personnel shortages, and the high number of households and the set passive structure, I could see the gap between services in urban and rural areas, and could sense the many problems and the absence of PHC*.

Participants stated, out of pocket payments could be reduced in some ways with the establishment of the FM project. Family physicians manage the patient’s treatment and prevent many parallel expenses in particular in specialist diagnosis tests. They also claimed that family physicians will improve the early detection of disease and prevent catastrophic expenditure of complicated state.

*“The timely diagnosis of the disease at the lowest level of people’s exposure to the health care system prevents much of the financial burden imposed on them. A family doctor can be of great help in making an early diagnosis*”.

*“A family doctor dose not merely has a pure therapy perspective, but he/she can prevent the diseases that may threat people later”*.

### 3.3 The Management of Chronic Diseases

Another aspect that physicians thought the family medicine could complement was the reinforcing of the current PHC model against the growing burden of non-communicable diseases and chronic condition. They argue the current passive primary care in urban area cannot answer to high burden of chronic diseases and there is a pressing need for implementation of family medicine as a means for arming the current health network against disease profile.

“Our patients are currently treated incompletely, there is no follow-up, and this is due to the delay in implementing of the project. I say this project should be done 10 years ago”

“Again, I emphasize, here (a family physician office), if a patient come in and we failed to solve his problem, we advise him that you get what channel for treatment. Because he is our patients and will return” (a family physician in pilot provinces)

“This project allows the patients and doctor work together and has responsibility”

## 4. Discussion

The World Organization of Family Doctors (Wonca) describes the practices/roles that a family physician can perform in general. But, in fact, the characteristics of the health system in which family medicine working, determine actual roles in practice (Ssenyonga & Seremba, 2007).The present study investigated family medicine in the urban population of Iran and identified the key roles that it can play in response to current and future challenges of Iran health care system. Given the comprehensive nature of the study in capturing the perspectives of decision-makers in main organizations in different level of health system (the Ministry of Health, insurance companies, medical universities, NGOs and the local health network), results of the study contain information for policy-makers in Iran as well as in other countries seeking to establish family medicine in health systems.

Although development of the referral system is a legal requirement of national development plans of Iran (Moghaddam et al., 2013), but the lack of a referral system and poor PHC coverage in urban areas, particularly in the outlying area, still counts as a shortcoming of the health system in Iran (Moghadam et al., 2012). Our participants emphasized on the vast capacity of family medicine for developing a referral system in urban areas. In the meanwhile, Iran has had the experience of implementing a family medicine and referral system integrated with rural insurance in its rural areas; though the particular implementation style of this integrated plan hindered its optimal execution (Takian et al., 2011).

Our participants noted the low status of general practice against the current specialization-oriented health care demands. They also reported that general practitioners are denied of their rightful status against specialists and considered the implementation of this project as an opportunity to revive the true value of general practice and general practitioners. In concordance with these findings, in UK, the integration of general practice with PHC led to the enhancement of general practice in aspects such as professional status, the extent and type of care provided, financial incentives and formal postgraduate education; in addition, it highly benefited the NHS (Bradley & David McKelvey, 2005; Goodwin et al., 2011).

World Health Organization (WHO) (2000) considered responsiveness as one of the three main objectives of health systems. According to the our respondents, the lack of a named director in health system to guides patients for seeking their objective needs leads to people faced with low levels of responsiveness. Nevertheless, they believed that assigning a family physician to a certain population will improves accountability and responsiveness to the patients. In concordance with these statements are the findings Tähepőld et al. (2006) in Estonia. The researchers studied Patient expectations from consultation with family physician. They found high family physician’s overall responsiveness to patient (in Two-thirds of patients).

Results of the present study indicate that providing PHC through FM improves the patient’s financial and geographical access particularly in the outlying area. During interviews, participants reported that, in spite of the developments and achievements of PHC in rural areas of Iran, the PHC model is not well developed in urban areas and requires to be redesigned for the revival of the current health network. Maeseneer and Maaike (2010) investigated the family physician fitness in PHC in Africa. They concluded that well-trained family physicians can contribute to provide the fundamental right of access to quality primary health care for all citizens.

While high out of pocket payments are considered a major challenge posed to Iran’s health system (Ibrahimipour et al., 2011; Rajabi et al., 2013), our participant believed the health expenses imposed on people are partially due to unnecessary care and repeated specialized interventions. They expected family physician as a guiding agent for patients can prevent the imposition of these fees. They also believed commitment of FPs to their population health help to detection diseases in early stage lead to prevent of high cost of complicated stages. In concordance with these statements, Chetty et al. (2011) studied the effects of FPs on readmission rates and cost. Authors concluded that increasing the numbers of FPs is associated with significant reductions in hospital readmissions and considerable cost savings.

Many previous studies have indicated that disease transition and high burden of non-communicable diseases is a current and future critical challenge of Iran health system (Rajabi et al., 2013; Khajehkazem et al., 2005). According to our participant statements, current passive primary care in urban area cannot answer to high burden of chronic diseases and there is a pressing need for a strenuous model. In addition, they asserted to the preventive roles of family physicians, collaboration with specialist and long engagement with patient’s condition will lead to better management of chronic conditions. In similar with these statements, Sepanlou (2010) has claimed that Iran’s health system is not properly equipped to cope with the rapid expansion of chronic diseases. The experience of diseases transition in other countries also faced them to the same challenges and forced them to the similar agenda. Ramli and Taher (2008) studied the management of chronic disease in the Malaysian PHC, they concluded the current PHC setting in Malaysia need to be redesigned to response the growing population of patients with chronic conditions.

## 5. Conclusion

The findings of our study indicated that policy-makers and decision-makers of the various organizations of Iran health systems consider family medicine as a viable means for a series of crucial reforms. In the face of the current health challenges, implementation of family medicine can strengthen the PHC model in Iran urban areas. Attempting to create a general consensus among various stakeholders is essential to the effective implementation of the project.
